# 58055 Mothers’ and Fathers’ Parent-Child Aggression Risk, Intimate Partner Violence, and Perceived Child Behavior Problems

**DOI:** 10.1017/cts.2021.727

**Published:** 2021-03-30

**Authors:** Doris F. Pu, Christina M. Rodriguez

**Affiliations:** University of Alabama at BIrmingham

## Abstract

ABSTRACT IMPACT: In light of the high co-occurrence between intimate partner violence (IPV) and physical child abuse, studying these forms of aggression simultaneously, bidirectionally, and longitudinally is vital to address this public health need. OBJECTIVES/GOALS: This study examined reciprocal associations between parent-child aggression (PCA) risk, IPV victimization, and perceived child behavior problems, to evaluate whether negative processes can transmit across family subsystems (i.e., spillover hypothesis) and/or across individuals (i.e., crossover hypothesis) over time. METHODS/STUDY POPULATION: Participants were first-time mothers and their male partners enrolled in a prospective longitudinal study, which tracked parenting and PCA risk over the transition to parenthood and into early childhood. The current project examined data from the third and fourth waves, when participants’ child was 18 months old and approximately 4 years old. At both timepoints, parents reported on their PCA risk (i.e., child abuse potential, harsh parenting), physical and psychological IPV victimization and perpetration, and perceived child behavior problems. Mothers and fathers each completed protocol on laptops in separate private rooms. Hypotheses were tested with autoregressive cross-lagged path models, which were estimated for mothers and fathers separately as well as dyadically. RESULTS/ANTICIPATED RESULTS: Findings partially supported the hypotheses, with evidence of spillover occurring bidirectionally for mothers and unidirectionally for both mothers and fathers. Mothers’ PCA risk predicted their subsequent IPV victimization and their reported child behavior problems (i.e., spillover effects) as well as fathers’ reported IPV victimization (i.e., crossover effect). Maternal reports of child behavior problems also predicted mothers’ reported IPV victimization and fathers’ reported child behavior problems, indicating possible child-driven effects. Overall, results demonstrate the mutual influence of individuals and subsystems within the family. Additionally, mothers rather than fathers appear more vulnerable to harmful spillover effects. DISCUSSION/SIGNIFICANCE OF FINDINGS: The need for family prevention and intervention services is clear, given the complex, transactional nature of family violence. Particularly for mothers, higher PCA risk may herald an increased risk for subsequent IPV victimization and vice versa. Clinical implications for parent-focused intervention programs are discussed.

